# Apparent vitamin D intoxication due to paraprotein interference

**DOI:** 10.1210/jcemcr/luag297

**Published:** 2026-09-23

**Authors:** Sjaam Jainandunsing, Evert F S van Velsen, Pauline Verschuure, Dennis Willemsen

**Affiliations:** Department of Internal Medicine, Anna Hospital, Geldrop 5664 EH, The Netherlands; Department of Internal Medicine, Erasmus MC, Rotterdam 3000 CA, The Netherlands; Department of Clinical Chemistry, Anna Hospital, Geldrop 5664 EH, The Netherlands; Department of Internal Medicine, Anna Hospital, Geldrop 5664 EH, The Netherlands

**Keywords:** hypercalcemia, vitamin D, multiple myeloma, paraprotein interference, immunoassay, LC-MS/MS

## Abstract

A 76-year-old man presented shortly after knee arthroplasty with malaise, nausea, and severe parathyroid hormone–independent hypercalcemia. Initial laboratory evaluation demonstrated a markedly elevated 25-hydroxyvitamin D concentration (>250 nmol/L [>100 ng/mL]; reference >50 nmol/L [>20 ng/mL]), suggestive of vitamin D intoxication. However, this diagnosis appeared implausible because the patient used only 400 IU cholecalciferol daily, serum phosphate remained normal, and several Calcium elevation, Renal dysfunction, Anemia and Bone disease features suggested an underlying plasma cell disorder. Further evaluation confirmed symptomatic immunoglobulin A kappa (IgA-κ) multiple myeloma. Repeat measurement of 25-hydroxyvitamin D by liquid chromatography–tandem mass spectrometry (LC-MS/MS) demonstrated a normal concentration (92 nmol/L [36.8 ng/mL]), confirming analytical interference. The falsely elevated result was reproduced in an independent reference laboratory, and 25-hydroxyvitamin D normalized following successful antimyeloma therapy in parallel with a marked reduction in paraprotein concentration. This case highlights the importance of integrating laboratory findings with clinical reasoning and confirms the value of LC-MS/MS in patients with clinically implausible vitamin D concentrations.

## Introduction

Hypercalcemia is a common metabolic abnormality encountered in endocrine and internal medicine practice. Following demonstration of suppressed parathyroid hormone (PTH), the differential diagnosis includes malignancy-associated hypercalcemia, vitamin D–mediated hypercalcemia, granulomatous disease, medication-related causes, and immobilization [1]. Measurement of serum 25-hydroxyvitamin D (25[OH]D) therefore frequently forms part of the diagnostic evaluation.

Most clinical laboratories measure serum 25(OH)D using automated immunoassays because of their rapid turnaround time and broad availability. Despite their widespread use, immunoassays remain vulnerable to analytical interference. Monoclonal immunoglobulins are well-recognized causes of assay interference and may affect immunochemical measurements through several mechanisms including nonspecific binding, matrix effects, increased viscosity, aggregate formation, and interference with reagent binding [2]. However, analytical interference causing falsely elevated serum 25(OH)D concentrations has only rarely been described. Previous reports primarily involved patients with monoclonal gammopathies in whom confirmation by liquid chromatography–tandem mass spectrometry (LC-MS/MS) established the correct serum 25(OH)D concentration [3-5]. We report a patient with immunoglobulin A kappa (IgA-κ) multiple myeloma presenting with apparent severe vitamin D excess during evaluation of PTH-independent hypercalcemia, illustrating how careful clinical reasoning prevented diagnostic error.

## Case presentation

A 76-year-old man was admitted 12 days after elective right knee arthroplasty because of progressive malaise, nausea, and poor oral intake. His medical history was unremarkable with regard to endocrine disorders. He reported no use of over-the-counter supplements other than cholecalciferol 400 IU daily.

## Diagnostic assessment

On physical examination, the patient was alert and did not appear acutely ill. Blood pressure was 168/91 mmHg, heart rate 108 beats/minute, respiratory rate 13 breaths/minute, oxygen saturation was 96% on room air, and body temperature was 37.0 °C. Cardiac and pulmonary examinations were unremarkable, and the abdomen was soft and nontender. The surgical wound of the right knee was mildly warm with some hematoma formation, without clinical signs of infection or wound discharge.

Initial laboratory evaluation demonstrated severe albumin-corrected hypercalcemia (3.48 mmol/L [13.9 mg/dL]; reference, 2.15-2.55 mmol/L [8.6-10.2 mg/dL]) together with a suppressed PTH concentration (0.39 pmol/L [3.7 pg/mL]; reference, 1.8-7.8 pmol/L [17-74 pg/mL]), indicating PTH-independent hypercalcemia (Table 1). Surprisingly, serum 25(OH)D measured using the Cobas Vitamin D Total III assay (Roche Diagnostics) exceeded 250 nmol/L (>100 ng/mL; reference, >50 nmol/L [>20 ng/mL]) on routine laboratory testing.

**Table 1:** Laboratory findings at presentation and after treatment

Laboratory parameter	Presentation, SI (conventional)	After treatment, SI (conventional)	Reference interval, SI (conventional)
Corrected calcium	3.48 mmol/L (13.9 mg/dL)	2.23 mmol/L (8.9 mg/dL)	2.15 to 2.55 mmol/L (8.6-10.2 mg/dL)
Albumin	26 g/L (2.6 g/dL)	34 g/L (3.4 g/dL)	35 to 50 g/L (3.5-5.0 g/dL)
Parathyroid hormone	0.39 pmol/L (3.7 pg/mL)	—	1.8 to 7.8 pmol/L (17-74 pg/mL)
Hemoglobin	4.0 mmol/L (6.4 g/dL)	5.9 mmol/L (9.5 g/dL)	8.5 to 11.0 mmol/L (13.7-17.7 g/dL)
Mean corpuscular volume	103 fL	100 fL	80 to 100 fL
Total protein	120 g/L (12.0 g/dL)	66 g/L (6.6 g/dL)	63 to 83 g/L (6.3-8.3 g/dL)
Creatinine	129 μmol/L (1.46 mg/dL)	58 μmol/L (0.66 mg/dL)	64 to 104 μmol/L (0.72-1.18 mg/dL)
Estimated glomerular filtration rate	46 mL/min/1.73 m^2^	>90 mL/min/1.73 m^2^	>60 mL/min/1.73 m^2^
Phosphate	1.00 mmol/L (3.10 mg/dL)	—	0.80 to 1.50 mmol/L (2.5-4.6 mg/dL)
Erythrocyte sedimentation rate	>140 mm/hour	79 mm/hour	<20 mm/hour
IgA-κ monoclonal protein	75 g/L	10.54 g/L	Not detected
25-Hydroxyvitamin D (automated immunoassay)	>250 nmol/L (>100 ng/mL)	81 nmol/L (32.4 ng/mL)	>50 nmol/L (>20 ng/mL)
25-Hydroxyvitamin D (LC-MS/MS)	92 nmol/L (36.8 ng/mL)	—	>50 nmol/L (>20 ng/mL)

Abbreviations: LC-MS/MS, liquid chromatography–tandem mass spectrometry; SI, International System of Units.

At first glance, these biochemical findings appeared compatible with vitamin D intoxication. However, several aspects of the clinical presentation were inconsistent with this diagnosis. The patient used only a physiological maintenance dose of vitamin D, denied additional supplementation, and serum phosphate remained within the normal reference range despite marked hypercalcemia. Moreover, laboratory testing simultaneously demonstrated severe anemia (hemoglobin 4.0 mmol/L [6.4 g/dL]; reference, 8.5-11.0 mmol/L [13.7-17.7 g/dL]), renal impairment (estimated glomerular filtration rate 46 mL/min/1.73 m^2^; reference, >60 mL/min/1.73 m^2^), and an elevated erythrocyte sedimentation rate (>140 mm/hour; reference, <20 mm/hour). Although these abnormalities were not explained by vitamin D intoxication, they raised suspicion of an alternative diagnosis.

Subsequent evaluation demonstrated marked hyperproteinemia (total protein 120 g/L [12.0 g/dL]; reference, 63-83 g/L [6.3-8.3 g/dL]), prompting serum protein electrophoresis, which identified an IgA-κ monoclonal protein of 75 g/L. Whole-body computed tomography revealed multiple osteolytic lesions. Bone marrow biopsy demonstrated approximately 80% clonal plasma cells with κ light-chain restriction, establishing the diagnosis of symptomatic IgA-κ multiple myeloma [6].

Because the markedly elevated serum 25(OH)D concentration remained physiologically implausible, an aliquot of the same original sample was remeasured using LC-MS/MS, which demonstrated a normal concentration of 92 nmol/L (36.8 ng/mL). This excluded vitamin D intoxication and established analytical interference as the explanation for the initial result.

To exclude a laboratory-specific analytical error, the original sample was analyzed in an independent reference laboratory using the same automated 25(OH)D immunoassay (Cobas Vitamin D Total III; Roche Diagnostics). Again, markedly elevated concentrations were obtained (>300 nmol/L [>120 ng/mL]; 358 nmol/L [143.2 ng/mL] after dilution), demonstrating that the interference was reproducible across laboratories.

## Treatment

Initial treatment of the severe hypercalcemia consisted of intravenous hydration with 3 L of 0.9% sodium chloride over 24 hours and a single intravenous dose of pamidronate 90 mg.

Subsequently, myeloma-directed therapy was initiated with daratumumab 1800 mg subcutaneously once weekly for weeks 1 to 8, every 2 weeks for weeks 9 to 24, and every 4 weeks thereafter; lenalidomide 25 mg orally once daily on days 1 to 21 of each 28-day cycle; and dexamethasone 20 mg orally once weekly. After 2 treatment cycles, the monoclonal protein concentration declined by more than 90% (75-10.54 g/L).

## Outcome and follow-up

Repeat serum 25(OH)D measurement using the same automated immunoassay now showed a concentration of 81 nmol/L (32.4 ng/mL), paralleling the reduction in paraprotein burden and strongly supporting paraprotein-mediated analytical interference.

## Discussion

The present case illustrates how analytical interference may fundamentally alter clinical reasoning during evaluation of PTH-independent hypercalcemia. At presentation, the biochemical profile appeared internally consistent: severe hypercalcemia, suppressed PTH, and markedly elevated serum 25(OH)D suggested vitamin D intoxication. However, careful consideration of the clinical context revealed several inconsistencies that prompted further investigation and ultimately prevented diagnostic error.

Vitamin D intoxication is an uncommon cause of hypercalcemia and generally results from prolonged ingestion of supraphysiological doses of vitamin D. Besides hypercalcemia and suppressed PTH, patients typically exhibit hyperphosphatemia due to increased intestinal calcium and phosphate absorption [1]. Our patient used only a maintenance dose of cholecalciferol (400 IU/day), denied additional supplementation, and phosphate concentrations remained within the normal reference range. These findings were difficult to reconcile with the apparently extreme serum 25(OH)D concentration reported by immunoassay.

Conversely, the simultaneous presence of anemia, renal impairment, marked hyperproteinemia, and hypercalcemia strongly suggested an underlying plasma cell disorder. Recognition of these Calcium elevation, Renal dysfunction, Anemia and Bone disease (CRAB) features shifted the diagnostic focus toward multiple myeloma despite the misleading vitamin D result. This sequence illustrates an important principle in endocrine diagnostics: laboratory results should always be interpreted within their physiological and clinical context.

Analytical interference caused by monoclonal proteins has been described in numerous laboratory assays for different lab measurements. Proposed mechanisms include nonspecific protein interactions, altered sample viscosity, aggregate formation, matrix effects, and interference with reagent binding [2]. A recent comprehensive review further proposed that serum 25(OH)D measurements may be affected through formation of immune macrocomplexes or incomplete dissociation of vitamin D from vitamin D–binding protein during the initial assay step [4, 7]. Although the precise mechanism responsible in our patient cannot be established, the disappearance of interference following effective myeloma treatment strongly supports a causal relationship between paraprotein burden and assay performance. The clinical consequences of paraprotein-associated analytical interference extend beyond vitamin D measurements. For example, paraprotein-induced pseudohypercalcemia has previously been reported [8], illustrating that monoclonal proteins may interfere with laboratory interpretation through different mechanisms and potentially lead to diagnostic errors.

Only a limited number of reports have described paraprotein-associated interference in serum 25(OH)D measurements. Ong et al [3] first reported artifactually elevated serum 25(OH)D concentrations in a patient with IgG multiple myeloma [3], while Hager et al [4] subsequently demonstrated similar findings in several patients with monoclonal gammopathies. In those reports, confirmation by LC-MS/MS consistently demonstrated normal serum 25(OH)D concentrations, emphasizing the importance of confirmatory testing whenever laboratory results appear physiologically implausible.

Our case adds several novel observations to the existing literature on laboratory measurements of serum 25(OH)D. First, the analytical interference was reproduced in 2 independent laboratories, excluding local analytical error. Second, interference diminished in parallel with a greater than 90% reduction in monoclonal protein concentration following antimyeloma therapy, providing strong biological evidence that the paraprotein itself was responsible for the falsely elevated result. Most previously reported cases involved the Abbott Architect 25(OH)D immunoassay, whereas our patient was evaluated using a different automated immunoassay platform [4]. This case therefore suggests that clinically relevant interference may not be confined to a single immunoassay platform and may occur across multiple automated serum 25(OH)D immunoassays. Whether IgA paraproteins are particularly prone to causing serum 25(OH)D assay interference remains uncertain. Although polymeric IgA molecules theoretically possess greater potential for aggregation than IgG, currently available evidence does not support preferential interference by a specific immunoglobulin isotype.

The principal clinical lesson extends beyond laboratory medicine. Had the initial serum 25(OH)D concentration been accepted uncritically, hypercalcemia might have been attributed to vitamin D intoxication, potentially delaying recognition and treatment of symptomatic multiple myeloma. More broadly, this case emphasizes that analytical interference should be considered whenever immunoassay-based laboratory results are discordant with the clinical presentation. In such situations, confirmation using an alternative analytical method should be considered.

This case demonstrates that monoclonal paraproteins may produce clinically significant analytical interference in automated 25(OH)D immunoassays, potentially mimicking severe vitamin D intoxication during the evaluation of PTH-independent hypercalcemia. Careful clinical reasoning, recognition of physiologically inconsistent laboratory findings, and confirmation using LC-MS/MS prevented diagnostic error and facilitated timely recognition of the underlying disorder.

## Learning points

- Markedly elevated serum 25(OH)D concentrations should always be interpreted within the clinical context.
- Analytical interference should be suspected when biochemical findings are physiologically implausible.
- Monoclonal gammopathies may produce falsely elevated serum 25(OH)D concentrations in automated immunoassays.
- Confirmation by LC-MS/MS is recommended when serum 25(OH)D results are discordant with the clinical presentation.
- Hypercalcemia in patients with CRAB features should prompt evaluation for plasma cell disorders even when vitamin D concentrations appear markedly elevated.

## Data Availability

Data sharing is not applicable to this article because no datasets were generated or analyzed during the current study.
